# Integration of Artificial Intelligence Into a Medical Curriculum: Evolving Student Perceptions and Faculty Development Challenges

**DOI:** 10.7759/cureus.109621

**Published:** 2026-05-25

**Authors:** Kinner L Flaglor, Gabrielle Rueff, Paul Monaco, Antonio E Rusinol, Mark Hernandez

**Affiliations:** 1 Medical Education, East Tennessee State University Quillen College of Medicine, Johnson City, USA; 2 Medical Education and Simulation, East Tennessee State University Quillen College of Medicine, Johnson City, USA; 3 Biomedical Sciences, East Tennessee State University Quillen College of Medicine, Johnson City, USA

**Keywords:** curriculum, faculty development, generative ai, medical education, teaching technology

## Abstract

Background and methods: The rapid integration of generative artificial intelligence (AI) into medical education presents both opportunities and continuing challenges for faculty and learners alike. This study provides an update on ongoing efforts to integrate AI into the curriculum at Quillen College of Medicine, East Tennessee State University in the United States, focusing on evolving student perceptions and a structured faculty development initiative. Using our previously published satisfaction instrument, we tracked shifting attitudes toward AI among our pre-clerkship and clerkship students.

Results: Incoming students demonstrated increasing awareness of AI's impact on education and the profession, alongside a stronger commitment to developing core clinical competencies independent of technology. The response rate for AI use was higher in 2026 compared to 2025 (97%, n=68 vs. 89%, n=62) for rising third-year students (class sizes of 80). Those students showed a meaningful shift toward using AI for active learning, particularly practice question generation and self-testing (Mean 2.30, SD 1.10 for 2025; Mean 2.77, SD 1.14 for 2026), while upper-level clerkship students consistently prioritized the ability to function as competent clinicians without AI assistance. To address faculty readiness, we developed a multi-dimensional framework for responsible AI integration and implemented a series of faculty development workshops. The first workshop produced an increase in confidence (+0.75) and practical engagement (+0.43). A second workshop focused on ethical and policy considerations yielded greater critical awareness, though with a more cautious outlook. Feedback across both workshops consistently highlighted the need for hands-on training, prompt engineering instruction, and role-specific, tool-agnostic development pathways.

Conclusions: Our findings underscore that integration of AI into medical education requires sustained, flexible, and mission-aligned faculty development. As AI becomes standard in clinical practice, equipping both educators and learners with the skills for responsible, thoughtful AI use is not optional; it is essential.

## Introduction

Large language models (LLMs) have already transformed health professions education. As more universities embrace generative artificial intelligence (AI), more than half of all college and medical students reported using these tools in 2025 [1-3]. Between 2024 and 2025, learners’ perceptions of AI shifted from cautious hesitancy toward broader acceptance of the technology [4,5]. However, rapid adoption and continuous exposure to AI tools have also heightened concerns about how medical students are using AI and whether they are fully developing the skills necessary for the successful practice of medicine [6].

Based on publicly available Association of American Medical Colleges (AAMC) survey instruments and national reports, the Post-Medical College Admission Test (Post-MCAT) Questionnaire [7], the Matriculating Student Questionnaire [8], the Year Two Questionnaire [9], and the Graduation Questionnaire [10] do not include explicit items assessing students’ use of AI tools. Emerging concerns among educators and learners range from the erosion of foundational skills to vulnerabilities that may arise when AI tools are unavailable, underscoring the need for rapid mitigation strategies. These strategies must support continued modernization and the full integration of AI [11,12]. This is particularly relevant in our medical school since we recently introduced a new curriculum and have been making minor modifications due to the challenges since the introduction of AI [13]. The process of AI modernization requires most, if not all, faculty to rapidly develop AI proficiency, ideally before current and future learners enter medical school. A lack of adequate knowledge about how to use AI tools effectively to support learning is a challenge for faculty [14], especially as some learners may now turn to chatbots instead of professors, textbooks, or session materials for guidance [15].

Rueff et al. have previously shown that medical schools can develop and implement plans to integrate generative AI into preclinical curricula while maintaining academic integrity and ethical standards [4]. Given the rapid rise in faculty and student adoption of AI and the evolution of related professionalism policies, the need to incorporate structured AI training for faculty in both the basic and clinical sciences is urgent. For a medical school with limited resources that has implemented a new curriculum, the central challenge lies in educating both learners and faculty to use AI effectively, ethically, and responsibly. Given the rapid rise in faculty and student adoption of AI and the evolution of related professionalism policies, we present an update on this ongoing transformation by: (i) highlighting our sustained efforts to encourage and train educators in the use of AI, and (ii) preparing preclinical students for the challenges of AI adoption during their clerkship training.

## Materials and methods

This was a study conducted at the Quillen College of Medicine at East Tennessee State University, Johnson City, Tennessee, United States. The study was approved by the East Tennessee State University Campus Institutional Review Board (approval number: c0524.9e-ETSU (IRB)).

In the Spring semester (2025) for current students (M2s) and in the Fall semester for incoming M1 students (2025), and again in the Spring semester for current M2/rising M3 students (2026), a satisfaction instrument previously developed by the authors [4,16] was distributed to assess current AI usage patterns and perceptions of AI technology in medical education. Faculty members were also invited to participate in the Spring and Summer 2025 administrations to capture educator perspectives alongside student responses. The surveys were distributed electronically via the Qualtrics platform (Qualtrics LLC, Provo, Utah, United States).

For each learning item, the mean and standard deviation (SD) were calculated to establish baseline ratings for each academic year and student level. Sample size fluctuated due to survey skip logic on Qualtrics: "Only students who identified as active AI users were prompted to rate specific study tools".

Data were analyzed using Microsoft Excel (Microsoft Corporation, Redmond, Washington, United States). Descriptive statistics, including means and standard deviations, were calculated for all learning items across cohorts. To identify shifts in student perspectives (class sizes of 80), inferential statistics were performed using independent sample t-tests. Given the unequal sample sizes between years, a two-sample t-test assuming unequal variances was utilized. Statistical significance was set at p < .05.

Following review of the survey feedback, and based on our previous published findings [4], our faculty adopted a multi-dimensional framework for AI integration encompassing institutional policy review, ethical disclosure practices, and tool-specific implementation strategies. This framework served as the foundation for a faculty roadmap for responsible AI integration and curriculum development, which in turn guided the objectives for our AI workshop series.

The first faculty development workshop, “Informing on AI tools to Optimize Teaching,” was held in August 2025 and focused on the effective use of AI tools within our educational environment. Presenters included representatives from academic technology services at our main university campus, the medical library, a medical student actively engaged in medical education research, and a national AI consultant. The workshop was primarily designed for teaching faculty and ran approximately four hours. Following the workshop, the authors convened to evaluate its impact on faculty views and confidence in the use of AI tools (Table 1).

**Table 1:** Faculty framework for responsible AI integration and curriculum development. Reflective summary by the authors of actionable items and objectives gathered after evaluations of the first AI workshop (four-hour duration), and based on the responses (N=40 for in-person participation). The workshop was available in-person and also via Zoom (Zoom Video Communications, Inc., San Jose, California, United States), and the recordings/materials from the workshop were made available to all faculty. The authors constructed this table after reviewing the transcript of the workshop to help evaluate its overall impact and the indirect potential impact on our curriculum. AAMC: Association of American Medical Colleges; AI: artificial intelligence

Category	Actionable Items and Objectives from August Workshop	Covered fully	Covered partially	Minimally covered
Policy and Compliance	Examples: 1) to review institutional AI guidance and AAMC resources, 2) to implement a framework for responsible implementation, or 3) identify short-term concerns and long-term strategic foci.		*	
Ethics and Transparency	Examples: 1) to adhere to core principles (transparency, human oversight, and privacy), 2) to disclose AI use in syllabus and for all course materials/grading, or 3) to turn off training data options when handling sensitive information.	*		
Instructional Quality	Examples: 1) to verify accuracy and check for bias in all AI-generated content, 2) to establish human-review mechanisms for disrupted AI assessments, or 3) to provide examples for student education on AI limitations and environmental impacts.	*		
Technical Integration	Examples: 1) to explore Amboss AI tools (GPT, Browser Extension) for lesson plans and learning management system (LMS) integration, 2) to utilize AI tools (e.g., ChatGPT (OpenAI Group PBC, San Francisco, California, United States) or Claude (Anthropic, San Francisco, California, United States)) for brainstorming and content polishing, or 3) to develop guidelines for AI use in research and/or Problem-Based Learning (PBL).	*		
Assessment and Research	Examples: 1) to use AI-powered search for tailored assessments and remediation, 2) to monitor research on AI’s impact on critical thinking via curated PubMed searches, or 3) to evaluate resource needs and potential investment based on usage data.			*

Evaluation of the feedback from the August workshop suggested more training was needed, with a focus on the ethical use of AI (copyright issues, plagiarism, etc.). A second workshop, “Beyond the Hype: Ethical and Policy Frontiers of AI in Biomedical Research and Publishing,” focusing on risk awareness, was held in December 2025. The second workshop was open to faculty and biomedical sciences graduate students. However, due to scheduling conflicts, faculty participation was more limited, and we believe that due to the timing late in the semester, the impact on the pre-clerkship curriculum was limited for current students. An AI tool (Claude Anthropic) was used to confirm the authors’ independent findings and evaluations.

## Results

As we explored the usage and perceptions of AI in the incoming classes for the Fall of 2024 and 2025, more students responded in 2025 (Table 2), and a higher number confirmed they used AI tools prior to starting medical school. Some incoming students in both cohorts reported using AI for testing understanding and flashcard generation, which suggests they had likely integrated AI into their self-study workflows. With a class size of 80, the limited sample sizes of 16 and 21 result in a relatively high margin of error in Table 2. Nevertheless, there is a clear increase in the perceived importance of learning about AI's impact on both education in 2025 (mean = 3.16) and on the profession (mean = 3.53). A higher value was placed on being able to function without AI (mean = 3.89) when comparing responses from 2024 to 2025. There was an increase in how much students value learning about AI's impact on their education (p=0.010) and their future profession (p=0.003), suggesting that the 2025 cohort was much more aware of the external pressures and changes AI is bringing to the medical field. While students were using AI tools more, their commitment to baseline medical competency independent of technology had strengthened (p=0.013). 

**Table 2:** Comparison of AI importance ratings between incoming M1 medical student cohorts (2024 vs. 2025). Data for categories related to professional Impact are from students who completed the survey and answered YES to: Do you use generative AI tools for learning/professional purposes at ETSU? Sample sizes (N) reflect the total number of students who provided ratings for each category. Scale: 1 = Not at All Important, 2 = Somewhat Important, 3 = Important, 4 = Very Important. Trend points toward growing reliance on AI for text-heavy tasks like summarization and self-testing (*statistically significant at p < 0.05). ETSU: East Tennessee State University; AI: artificial intelligence

Learning Item	2024 Mean (SD)	2025 Mean (SD)	t-statistic	p-value
Study Tools & Tasks	(N=5)	(N=15)		
To summarize learning materials	1.60 (0.55)	2.40 (1.06)	-1.604	0.126
To summarize assigned reading	1.80 (0.45)	2.73 (1.03)	-1.956	0.066
To create practice questions	1.80 (1.10)	2.53 (1.19)	-1.218	0.239
To schedule study plans	1.40 (0.55)	1.40 (0.74)	0	1
To develop mnemonics	1.60 (0.89)	1.53 (0.83)	0.155	0.879
To develop clinical cases	1.80 (1.10)	1.60 (0.63)	0.499	0.624
To test understanding	1.80 (1.10)	2.67 (1.23)	-1.412	0.175
To generate flashcards	1.80 (1.10)	1.93 (1.21)	-0.214	0.833
To create patient simulations	1.60 (0.89)	1.27 (0.46)	1.119	0.278
Help answering questions	1.20 (0.45)	1.53 (0.64)	-1.077	0.296
Professional Impact	(N=16)	(N=21)		
Prepared to work using AI	2.38 (0.87)	2.44 (0.62)	-0.237	0.814
Function as clinician with AI	2.31 (0.95)	2.61 (1.04)	-0.871	0.39
Function as clinician without AI	3.08 (1.26)	3.89 (0.32)	-2.631	0.013*
AI impact on education	2.46 (0.88)	3.16 (0.60)	-2.736	0.010*
AI impact on profession	2.69 (0.85)	3.53 (0.70)	-3.193	0.003*
Gaining clinical experiences w/ AI	2.23 (0.93)	1.94 (0.80)	0.963	0.342

**Figure 1:**
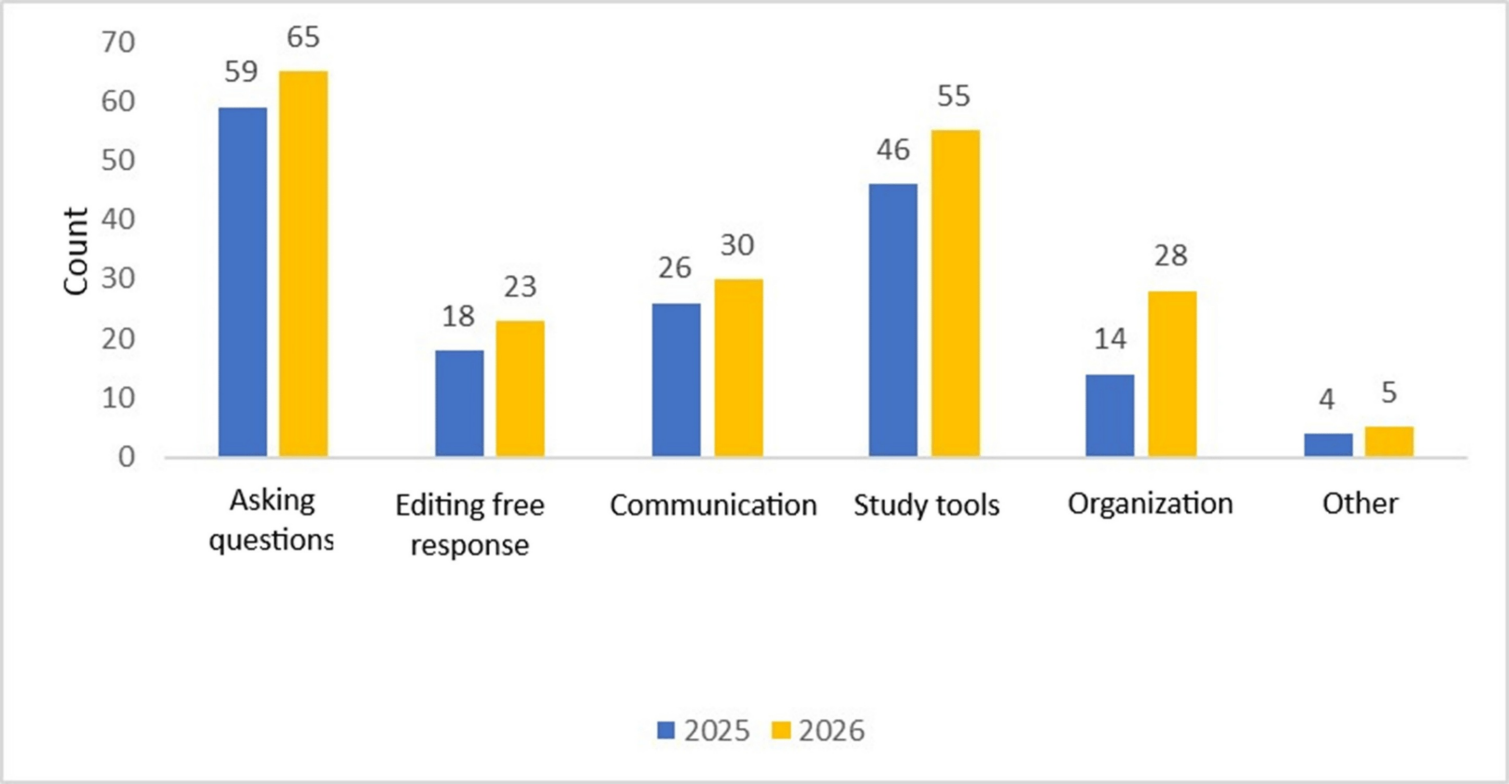
Comparison of AI usage among students entering clinical clerkships. Student reported use of AI at beginning their clinical clerkships (February 2025 and 2026).  Data represent the actual count from rising M3-students. The overall class size is 80 for each cohort. Students who completed the survey and answered YES to: Do you use AI tools for learning/professional purposes at ETSU? If yes, describe your usage (select all that apply) 1: asking questions as a search engine; 2: editing free response answers, essays, or assignments, 3: communication purposes (e.g., composing or editing emails), 4: study tools (e.g., summarize learning material, creating practice tests/questions, study plans, mnemonics, patient cases, etc.), 5: organization, creation, or presentation of lecture material for designated courses, and 6: other. For more information see Rueff et al., 2025 [4]. ETSU: East Tennessee State University; AI: artificial intelligence

As we examined the perspectives of the rising M3 (those incoming clinical training) students during their transition to the clinical clerkship course, the response rate was found to be higher: 97% (n=68) in 2026 compared to 89% (n=62) in 2025, and there was a shift in how AI tools are used (Figure 1).

Using AI for organization, creation, or presentation of lecture material for designated courses showed the greatest shift. Between 2025 and 2026, there was a noticeable increase in students using AI for active learning (Table 3). For example, students placed higher importance on using AI to create practice questions (p=0.016) and to test their understanding (p=0.004), suggesting they were moving away from just "reading summaries" toward using AI as a personal tutor. On the other hand, using AI to develop mnemonics declined (p=0.045), suggesting that students find other study methods more effective. While M3 and M4 students on their clerkship rotations in January 2026 found AI useful for summarizing materials and testing understanding, there were no differences between how students view AI in early vs later rotations (data not shown) and notably interest in using AI to write clinical notes (SOAP (subjective, objective, assessment, plan) notes/progress notes) remained relatively low for both cohorts (M3, mean = 1.77, SD 1.01; M4, mean 2.00, SD 1.33) likely reflecting current hospital policies or the specific nature of clinical rotations where students are still mastering traditional documentation. M3 students were becoming more concerned with how AI will impact their education (p=0.034) and their profession (p=0.002). 

**Table 3:** Comparison of AI importance ratings from rising M3 student cohorts during transition to clinical clerkships (January 2025 vs. January 2026). Students who completed the survey and answered YES to: Do you use generative AI tools for learning/professional purposes at ETSU? Sample sizes (N) reflect the total number of students who provided ratings for each category (some participants did not answer every question). Scale: 1 = Not at All Important, 2 = Somewhat Important, 3 = Important, 4 = Very Important (*statistically significant at p < 0.05). ETSU: East Tennessee State University; AI: artificial intelligence

Learning Item	Rising M3 2025 Mean (SD)	Rising M3 2026 Mean (SD)	t-statistic	P-value
Study Tools & Tasks	(N=66–68)	(N=72–73)		
To summarize learning materials	2.61 (1.10)	2.77 (0.95)	-0.889	0.375
To summarize assigned reading	2.52 (1.15)	2.71 (0.96)	-1.056	0.293
To create practice questions/vignettes	2.30 (1.10)	2.77 (1.14)	-2.45	0.016*
To schedule study plans	1.69 (1.00)	1.69 (1.03)	-0.046	0.964
To develop mnemonics	2.28 (1.14)	1.90 (1.05)	2.026	0.045*
To develop clinical cases	1.88 (0.98)	2.21 (1.11)	-1.848	0.067
To test my understanding	2.32 (1.20)	2.86 (0.99)	-2.895	0.004*
To generate flashcards	1.63 (0.92)	1.74 (0.98)	-0.679	0.498
To create patient simulations	1.66 (0.88)	1.81 (0.97)	-0.947	0.345
Professional Impact	(N=67–68)	(N=73)		
Being prepared to work using AI	2.85 (1.01)	3.03 (0.91)	-1.073	0.285
Functioning as clinician using AI	2.99 (0.94)	3.05 (0.98)	-0.429	0.668
Functioning as clinician without AI	3.25 (1.00)	3.53 (0.75)	-1.904	0.059
AI impact on education	2.75 (1.02)	3.08 (0.81)	-2.143	0.034*
AI impact on profession	2.90 (0.92)	3.34 (0.79)	-3.088	0.002*
Gaining clinical experiences w/ AI	2.75 (1.01)	2.78 (0.95)	-0.186	0.852

Workshops

By Spring 2025, several faculty members were already “ahead of the curve,” using AI to modify and develop new teaching materials. However, data from a faculty development workshop held in August 2025 indicated that other instructors were less confident in their use of AI. Notably, participants reported increased confidence following the workshop (Table 4).

**Table 4:** Opinions pre and post August 2025 faculty development workshop. This workshop "Informing on AI tools to Optimize Teaching" focused on skill acquisition. Confidence was measured on a 5-point scale, while objectives were measured on a 4-point scale. The workshop demonstrated how ChatGPT (OpenAI Group PBC, San Francisco, California, United States) could best be used for preparation of teaching materials. The confidence shift after the workshop (+0.75) is significant (p = 0.013). A notable upward shift (+0.43) in how clearly objectives were perceived during the workshop is noted.

Informing on AI tools to Optimize Teaching	Pre workshop		Post workshop		t-statistic	p-value	Trend Confidence
	Mean (N)	SD	Mean (N)	SD			
How would you rate your confidence using ChatGPT? 1 = Not at all confident, 2 = Slightly confident, 3 = Moderately confident, 4 = Quite confident, 5 = Extremely confident	2.72 (25)	1.17	3.47 (15)	0.64	-2.60	0.01	+0.75
Were the objectives of the workshop clearly defined and communicated before today’s workshop? 1 = Not at all, 2 = Slightly, 3 = Moderately, 4 = Extremely	3.21 (24)	0.78	3.64 (14)	0.63	-1.87	0.07	+0.43

As shown in Table 4, most workshop attendees recognized the importance of AI, although their levels of familiarity and actual use varied widely. Hesitation persisted earlier, particularly in early 2024, likely due to unclear institutional policies and ongoing mixed feelings about AI tools among faculty. Nevertheless, collective survey results from Spring and Summer 2025 highlighted a clear need for dedicated faculty development initiatives to support instructors in building confidence and engaging more openly and experimentally with AI technologies.

Feedback from the workshop in August 2025 informed the planning of a second workshop held in late Fall 2025. The overall sentiment following this second workshop presented a notably different pattern (Table 5). Rather than a boost in confidence, participants reflected a more cautious and critical stance, while still finding the workshop valuable. Attendees became more aware of the complexities and risks associated with AI use and expressed heightened expectations and greater concern for data privacy. The observed drop in confidence likely reflects the workshop's success in surfacing the limitations and pitfalls of AI, effectively moving participants from a state of unconscious incompetence to a more realistic, if more measured, understanding of the technology. 

**Table 5:** Opinions pre and post December 2025 faculty development workshop. This workshop “Beyond the Hype: Ethical and Policy Frontiers of AI in Biomedical Research and Publishing” focused on risk awareness and it included participation of graduate students. Confidence was measured on a 5-point scale, while objectives were measured on a 4-point scale. The confidence shift after the December workshop (-0.24) is not significant (p = 0.521). Note: Since our participants overwhelmingly reported use of ChatGPT (OpenAI Group PBC, San Francisco, California, United States) the survey items were not modified for this workshop. Although the workshop was found to be valuable, the confidence drop suggests that the session may have introduced complex topics that made the original objectives more difficult to answer.

Beyond the Hype: Ethical and Policy Frontiers of AI in Biomedical Research and Publishing	Pre workshop		Post workshop		t-statistic	p-value	Trend Confidence
	Mean (N)	SD	Mean (N)	SD			
How would you rate your confidence using ChatGPT? 1 = Not at all confident, 2 = Slightly confident, 3 = Moderately confident, 4 = Quite confident, 5 = Extremely confident	3.38 (16)	0.96	3.14 (7)	0.69	0.66	0.52	-0.24
Were the objectives of the workshop clearly defined and communicated before today’s workshop? 1 = Not at all, 2 = Slightly, 3 = Moderately, 4 = Extremely	3.25 (16)	0.77	3.14 (7)	0.38	0.45	0.66	-0.11

## Discussion

The data presented in Tables 2, 3 reflect a preliminary trend analysis examining the evolving attitudes of specific medical student cohorts during a period of rapid technological change. Given the voluntary nature of the surveys, these results do not represent a definitive census of student opinion. Nevertheless, despite modest participation numbers among the entering classes of 2024 and 2025, the findings are highly relevant to curriculum development as they offer a meaningful window into directional trends in student attitudes toward AI in teaching and learning. This relevance is strengthened by using identical survey instruments across cohorts, which supports internal consistency and allows for meaningful comparison over time.

Notably, the incoming 2025 cohort demonstrated considerably greater awareness of the external pressures and changes AI is bringing to the medical field (Table 2). This was not unexpected, but it reinforced the urgency of ensuring faculty had completed an AI workshop prior to the start of the Fall 2025 semester. Across entering cohorts, the data point toward a growing reliance on AI for text-heavy tasks such as summarization and self-testing, skills students are developing before they even begin medical school. For educators, understanding this shift is essential for designing learning activities that thoughtfully incorporate AI into the medical education process.

As these cohorts progressed through the pre-clerkship phase into the clerkship phase, rising third-year students expressed strong views about AI's impact on both their education and the profession, yet reported insufficient clinical experience to meaningfully evaluate AI use in that setting (Table 3). A concern that has been expressed in the literature [6] and among our students completing clerkship rotations (M3s and M4s) seems to be a clear and unified trend. Our learners ranked the ability to function as a competent professional without relying on AI as their highest priority (no statistical differences between M3s and M4s; data not shown). This suggests a shared conviction among upper-level students that core clinical skills must be developed and maintained independently of technology.

Through the development and use of our satisfaction instruments, we have gained valuable insight into how our learners' engagement with AI evolves across the curriculum, information that has directly informed faculty planning. Through our workshop series, we have learned that one size does not fit all. Flexible, ongoing faculty development is essential as our institution, like many others, moves beyond pilot projects toward meaningful, sustained integration of AI into the curriculum. Faculty varied widely in their familiarity with and use of AI tools, reflecting the diversity of roles and needs across our institution. Just as students benefit from flexible learning pathways to bridge the gap between AI's potential and clinical reality, faculty benefit from development tracks tailored to their specific professional contexts.

Drawing on research collaborations among students, researchers, and educators as well as consultations with AI experts and ethicists, we developed our workshop series to ensure that our approach to AI education and training remained meaningful and mission-aligned. Pre- and post-workshop surveys from the first workshop revealed a clear shift from cautious curiosity to increased confidence and practical engagement. Overall sentiment improved from a moderate baseline to a strong positive consensus, with post-workshop responses reflecting a faculty group that felt more capable and was actively planning how to integrate AI tools into their teaching and research. Not surprisingly, sentiment from the second workshop (Table 5) declined as faculty and graduate student attendees were more likely to consider the complexities and risks associated with using AI. 

The growth in confidence following the first workshop is significant (Table 4), as our goal was to equip educators with the skills needed to serve as effective facilitators, whether in problem-based learning (PBL), team-based learning (TBL), clinical integration sessions, or other instructional contexts. Six themes emerged from the workshop feedback and those are: (i) Hands-on application: the dominant request was a shift from learning about AI to using it, (ii) Prompt engineering: multiple participants requested specific instruction on prompt development and training in crafting effective prompts, (iii) Interactive sessions: participants wanted opportunities to bring their own devices, work in small groups, and complete practical exercises during sessions, (iv) One-on-one support: there was particular interest in applying AI tools to personal course materials, including tools (Amboss (https://www.amboss.com/int), etc.) that students already use, (v) Pedagogy and student use: participants expressed interest in how AI can bridge classroom technology with instructional practice, including its potential for individual student tutoring and personalized learning, and (vi) Topic fatigue: at least one participant felt that too much time was already being devoted to this topic and indicated they would only attend future sessions if the focus were highly specific, such as ethics or student-led presentations.

We have offered multiple formats for AI introduction and training, including support for attendance at free and subscription-based webinars and workshops sponsored by organizations such as the International Association of Medical Science Educators (IAMSE) and AAMC. However, not all faculty can participate in synchronous sessions, and clinical and basic science faculty face distinct scheduling and professional demands. On April 2, 2026, we organized a three-hour informative session for the Department of Internal Medicine as part of a Faculty Development Seminar titled “AI in Clinical Practice and Research Options”; although it was informative and well-received, more training is needed. Some of the authors have previously been invited to give webinars [17] or present workshops to a general audience (most recently in March 2026) through external collaborations. Based on recorded attendance and strong interest in these workshops, we believe more training is needed. For example, faculty have suggested that there is a clear need for asynchronous modules, more hands-on sessions, and role-specific training that is tool-agnostic, grounded in principles, and flexible approaches rather than tied to a single platform. Content should be tailored to the varied roles faculty hold across teaching, assessment, research, and clinical education. As our workshop offerings continue to evolve, accessibility and scalability must remain priorities, enabling faculty to engage at different depths and on their own timelines.

Our educators are actively shifting toward deliberate instruction in medical AI literacy and are developing AI-powered tools that support learning and Socratic engagement. Whether it is an app built with ChatGPT (OpenAI Group PBC, San Francisco, California, United States), a Gem created with Gemini (Google LLC, Mountain View, California, United States), or an Agent developed with Claude (Anthropic, San Francisco, California, United States), the way medical education is delivered is rapidly evolving. From handout development and meeting new federal accessibility guidelines to pedagogical design and assessment item creation, AI has had a meaningful impact, though it remains unclear whether overall faculty workload has increased, decreased, or simply shifted in form.

One motivating factor to become familiar with AI may be the potential to develop novel prompts for teaching and learning. It is worth noting that as of March 2026, works generated by AI from a human prompt cannot be copyrighted, as they were not created by a human [18]. Our learners are also becoming active participants in this process, creating AI-powered study tools and contributing to innovation alongside the technology itself. This matters especially as students enter clinical rotations, where AI is already embedded in professional practice as a standard of care. According to a recent American Medical Association (AMA) poll, 80% of physicians are already using AI in a professional context, with 92% reporting that they want more education and training on these tools, reflecting an impact felt by millions of patients daily [19]. The FDA recorded a historic number of approvals for AI-enabled medical devices in 2025, a figure expected to grow further in 2026 [20]. 

The influence of AI on medical education and its direct implications for clinical practice cannot be understated. Nearly a decade ago, it was proposed that one of the defining roles of the 2025 medical educator would be that of a technology adopter, someone fluent in selecting and applying appropriate tools to support learning [21]. That future has arrived. In 2025, OpenEvidence (OpenEvidence, Miami, Florida, United States) became the fastest-growing AI application for physicians in history [22], and many institutions, including ours, encouraged students to incorporate this tool into their learning [4]. Yet the risks of AI use lie not only in what these tools say, but in what they omit. As reported in studies examining the safe use of AI in clinical settings, a high proportion of AI-related harms stemmed from errors of omission: missed diagnostic tests, failure to refer patients, and neglected critical follow-ups, making it clear that AI use does not automatically equate to safety [23].

In January 2025, the Federation of American Societies for Experimental Biology (FASEB) issued a statement cautioning that AI has the potential to exacerbate existing problems and introduce new challenges, urging close collaboration among stakeholders [24]. As information becomes increasingly accessible through technology, it has been suggested that health professionals will be defined less by what they know and more by their ability to ask good questions, critically evaluate information, and maintain patient-centered vigilance, as articulated back in 2020 [25] after projections in the growth of medical knowledge doubling in days and not years [26]. The role of the medical educator has been evolving toward technology adoption long before the arrival of AI. This interpretation is consistent with recent cross-sectional higher education research ‎showing that educators’ perceived usefulness and effectiveness of generative AI are positively ‎associated with technology readiness and social influence, while AI-related anxiety may ‎hinder acceptance, highlighting the importance of both cognitive preparedness and ‎institutional context in technology adoption [27]. As the health professional's role continues to shift away from serving as a repository of facts and toward mastering higher-order skills like critical thinking, clinical judgment, and the compassionate vigilance essential for safe patient care, equipping our educators and learners with the skills for appropriate, thoughtful AI use becomes not optional, but essential.

Study limitations

Several limitations should be considered when interpreting these findings. First, all student and faculty surveys were voluntary, resulting in modest participation numbers (incoming class of 80 students (n=16 and n=21 in 2024 and 2025, respectively)), which limits statistical power and generalizability. Second, this study was conducted at a single medical school that has recently implemented a new curriculum. The first cohort of students in our new curriculum graduated on May 8, 2026. This may reduce transferability to larger or more established programs. Third, faculty workshop sample sizes were small, and attrition between pre- and post-workshop surveys further limits the robustness of those comparisons. Fourth, the second workshop's impact on student outcomes was difficult to assess given its timing late in the Fall 2025 semester. Finally, as AI tools and institutional policies are evolving rapidly, findings reflect a specific and narrow window of time that may not fully capture the current landscape by the time of publication.

## Conclusions

The rapid and continuous evolution of generative AI tools demands an ongoing commitment to faculty development to keep educators current, confident, and effective. The risks of inattention are significant, as neglecting AI in faculty training risks widening existing educational disparities and contributing to downstream health inequities. Many educators currently lack sufficient preparation to use generative AI effectively, let alone guide learners in its appropriate use. Persistent uncertainty around plagiarism detection, ethical boundaries, and acceptable academic practice further erodes professionalism and academic integrity when left unaddressed.

As institutions allocate resources toward AI integration, ensuring that faculty training is cost-effective and high-impact is essential. Spending without clear educational goals or measurable outcomes risks squandering both financial and human capital. The rapid AI-driven transformation of medical education is no longer a projection; it is the reality our educators must be prepared to navigate.
